# Extra-renal locations of the a4 subunit of H^+^ATPase

**DOI:** 10.1186/s12860-016-0106-8

**Published:** 2016-07-02

**Authors:** Zoe J. Golder, Fiona E. Karet Frankl

**Affiliations:** Department of Medical Genetics, University of Cambridge, Cambridge, UK; Cambridge Institute for Medical Research, Cambridge Biomedical Campus Box 139, Hills Road, Cambridge, CB2 OXY UK

**Keywords:** Proton pump, H^+^ATPase, a4 subunit, Tissue-restricted, Uterus, Yolk sac

## Abstract

**Background:**

Vacuolar-type proton pumps help maintain acid–base homeostasis either within intracellular compartments or at specialised plasma membranes. In mammals they are made up of 13 subunits, which form two functional domains. A number of the subunits have variants that display tissue restricted expression patterns such that in specialised cell types they replace the generic subunits at some sub-cellular locations. The tissue restricted a4 subunit has previously been reported at the plasma membrane in the kidney, inner ear, olfactory epithelium and male reproductive tract.

**Results:**

In this study novel locations of the a4 subunit were investigated using an *Atp6v0a4* knockout mouse line in which a LacZ reporter cassette replaced part of the gene. The presence of a4 in the olfactory epithelium was further investigated and the additional presence of C2 and d2 subunits identified. The a4 subunit was found in the uterus of pregnant animals and a4 was identified along with d2 and C2 in the embryonic visceral yolk sac. In the male reproductive tract a4 was seen in the novel locations of the prostatic alveoli and the ampullary glands as well as the previously reported epididymis and vas deferens.

**Conclusions:**

The identification of novel locations for the a4 subunit and other tissue-restricted subunits increases the range of unique subunit combinations making up the proton pump. These studies suggest additional roles of the proton pump, indicating a further range of homologue-specific functions for tissue-restricted subunits.

**Electronic supplementary material:**

The online version of this article (doi:10.1186/s12860-016-0106-8) contains supplementary material, which is available to authorized users.

## Background

The vacuolar type proton pump, or H^+^ATPase, is responsible for ATP dependent proton movement across membranes of intracellular organelles such as lysosomes where maintenance of pH is important for organelle function. In higher organisms such pumps are also found at the plasma membranes of specialised epithelial cells such as those of the kidney, inner ear, and male reproductive tract [1–3].

The pump consists of two functional domains: a V_1_ domain made up of subunits A-H where ATP hydrolysis provides energy for proton movement, and a membrane-anchored V_0_ domain containing subunits a-e where proton translocation takes place. The domains couple by an interface of subunits from both domains forming a central stalk with three peripheral stalks providing structural support [4]. The full pump complex functions by a rotor–stator mechanism where energy release from ATP hydrolysis causes rotation of the central stalk which in turn causes rotation of the V_0_ proteolipid ring which facilitates proton translocation [5].

In higher organisms a number of the subunits in both the V_1_ and V_0_ domains have multiple homologues encoded by different genes that have different expression patterns throughout the body. Specific subunit homologues are found at the plasma membrane in specialised cells such as some kidney tubular epithelial cells (intercalated cells), narrow and clear cells of the epididymis, and in the ruffled boarder of osteoclasts; these subunits are referred to as ‘tissue-restricted’. While there is a high degree of sequence and structural similarity between the subunit homologues, substitution between them does not always occur. The different combinations of subunits found in specialised locations are hypothesised to allow regulation of trafficking and activity [6].

The a-subunit is the largest in the V_0_ grouping and contains an N-terminal hydrophilic cytosolic domain, a central hydrophobic transmembrane region that crosses the membrane 6–8 times, and a small cytosolic C-terminal tail [7–9]. The central domain permits proton transport in conjunction with the c subunit proteolipid ring [10]. Four homologues of the a-subunit have been identified in mammals, which have differential tissue expression [11, 12]. a1 is expressed ubiquitously and a2 is expressed both in endosomes and in acrosomes of elongated spermatids [13]. a3 is found in Sertoli cells of the testis, osteoclasts, extra-retinal tissue of the eye and endocrine cells such as pancreatic β-cells [12–15] and a4 is found in the α-intercalated cells of the kidney, the inner ear, the male reproductive tract and eye [11, 14, 16, 17]. Defects in various a subunits have been associated with human disease; the a2 subunit with cutis laxa, [18] the a3 subunit with infantile malignant osteopetrosis [19] and defects in the B1 and a4 subunits with distal renal tubular acidosis (dRTA) [3, 20].

In an effort to understand more fully the importance of the a4 subunit and its role in dRTA, an a4 knockout mouse was successfully created using β-galactosidase as a reporter for the null gene. This model closely mirrors the human disease, with mice having a metabolic acidosis, hyperchloremia and hypokalemia but display earlier onset hearing loss [21]. During the characterization of this strain, it was noticed that a4 expression appeared to be wider than previously reported and the investigation into these novel locations forms the backdrop to this study.

## Results and discussion

### Olfactory system

#### β-galactosidase (β-gal) staining and immunostaining in embryonic *Atp6v0a4*^*+/−*^ olfactory epithelium

**Fig. 1:**
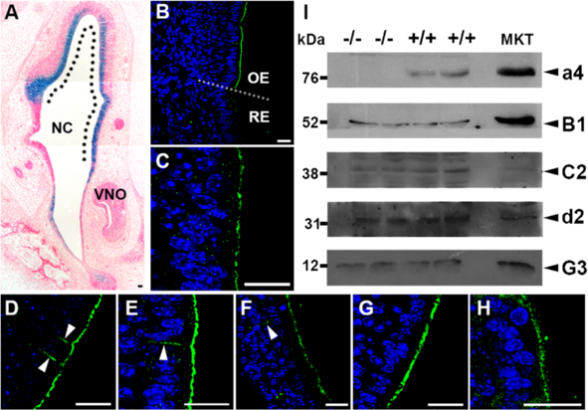
Coronal section of posterior nasal cavity (NC) and vomeronasal organ (VNO) at e16.5 stained for β-gal activity and eosin. The dotted line indicates the approximate location of the future olfactory epithelium (**a**). Apical membrane localisation of the a4 subunit in the OE in p5 animals and its absence in the RE (**b**). Apical membrane localisation of the F subunit in the OE of p5 animals (**c**). High powered images of OE showing apical and basolateral staining (arrow heads) of the B1 and a4 subunits at p5 and the F subunit at p30 respectively (**d**–**f**). The B1 and F subunit is maintained in an apical membrane location in −/− animals (**g**, **h**). Scale bars: 20 μm. Western blot analysis of total protein lysates from the OE of −/− and +/+ adult animals (40 μg) and total mouse kidney lysate (MKT) (20 μg) against various subunits of the proton pump (**i**). Sizes (kDa) are marked on the left with arrows marking the bands of interest on the right

Previous work examining whole-mount cleared embryos revealed that β-gal activity, which is driven by the a4 promoter in this model, was present in the nasal cavity from e12.5 onwards [21]. In the present study, sectioning of e16.5 embryonic heads revealed β-gal activity in a layer of cells lining the full length of the nasal cavity. Moving anterior to posterior along the cavity the staining becomes more specific to cells in the location of the future olfactory epithelium (OE) (Fig. 1a). Small amounts of β-gal activity were also seen in the vomeronasal organ (VNO) of 3 out of 3 samples examined. However, immunostaining of the OE and VNOs showed that neither the tissue restricted a4 nor B1 subunit protein could be detected at this stage of development, highlighting that promotor activity does not always equate to detectable protein expression. In contrast, the ubiquitously expressed F subunit was present in small amounts (not shown).

#### Immunostaining in postnatal olfactory epithelium

In the nasal cavity of p5 and p30 *Atp6v0a4*^+/+^ (+/+) animals, a4 immunostaining was absent from the squamous or respiratory epithelium (RE) but was present apically in OE (Fig. 1b) as previously observed [21]. Similar staining patterns were seen for the ubiquitous F subunit and tissue restricted B1 (Fig. 1c, d respectively). In addition, basolateral staining was seen in a subset of cells in the OE for B1, a4 and F subunits (white arrow heads Fig. 1d, e and f respectively) at both ages. These observations are consistent with published data of B1 and A subunit immunostaining [22] where positive staining was seen in a subpopulation of cells that were not olfactory sensory cilia. These cells were thought to be type II microvillar cells [22, 23], which may have a function as supporting cell precursors or modified sensory neurons [23]. In *Atp6v0a4*^−/−^ (−/−) animals of the same age, the staining in the OE for the B1 and F subunits was similar (Fig. 1g, h). The a subunit is essential for assembly of the V_0_ domain, which is in turn required for attachment to the V_1_ domain to form a functional pump [24, 25] suggesting that in this tissue, other a-subunit(s) are able to substitute for a4 and allow the pump to be assembled and trafficked to the membrane correctly. However, it was not possible to stain for the other a subunits due to lack of suitable a subunit antibodies. In VNO of p5 +/+ mice, a4, B1 and F were stained indicating existence of H^+^ATPases containing tissue-specific subunits in this location (Additional file 1: Figure S1A, B, and D respectively). Surprisingly however, in the −/− mice, B1 staining was absent in the VNO, although the F subunit was present (Additional file 1: Figure S1C and E respectively) and it is unclear why a4 ablation would affect B1 expression in this region.

#### Western blot analysis of expression of other tissue restricted subunits in olfactory epithelium

Protein lysates of adult OE were prepared from +/+ and −/− animals and analysed on western blots using several different antibodies. In addition to the tissue-restricted a4 and B1 subunits, C2, d2, and G3 subunits were also found (Fig. 1i), but their location could not be confirmed on tissue sections as the antibodies are not suitable for immunohistochemistry.

### The female reproductive tract

In humans, cervical mucus has a distinct ionic composition over the menstrual cycle [26] with a variable but acidic pH. How this acidification is achieved and regulated is unclear, with theories for both vaginal bacteria cohabitation [27] and proton secretion by vaginal epithelial cells [28] being advanced. In this study, the vaginal/cervical canal of +/− adult female mice had positive β-gal staining in cells of the stratified squamous epithelium that lines the cervical canal (Additional file 2: Figure S2A, B) suggesting expression of the a4 subunit. However, although RT-PCR showed that the a4 subunit was expressed, protein was not detected by immunostaining (Additional file 2: Figure S2C) or by western blot even though the ubiquitous D subunit was present. Immunostaining showed the presence, but no apical enrichment, of the ubiquitous F subunit (Additional file 2: Figure S2D) in the vagina/cervix suggesting that epithelial plasma membrane H^+^ATPases do not play a role in the acidification of the murine vaginal/cervical lumen.

**Fig. 2:**
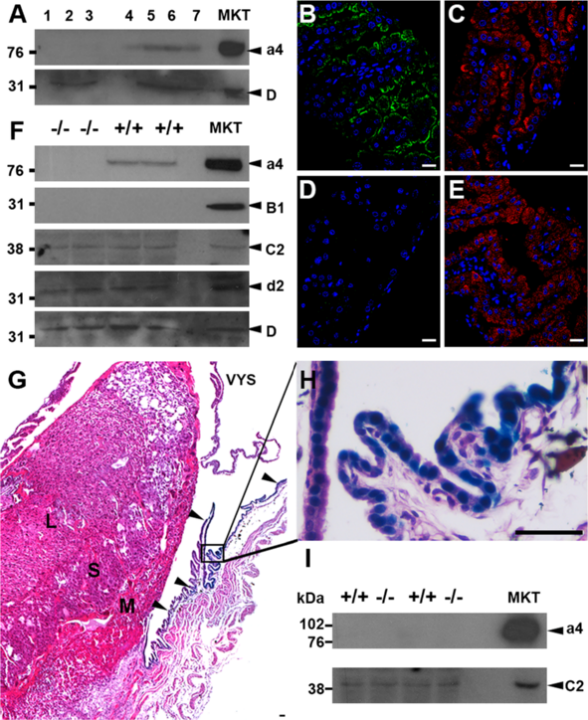
Female reproductive tract. Western blot of uterus lysates for the a4 and D subunits (**a**). Panels show uterus from +/+ non-pregnant mice (lanes 1–3), +/− uterus from around +/+ embryos (lanes 4, 6), +/− uterus from around −/− embryos (lanes 5, 7) (40 μg) and total mouse kidney (MKT) (20 μg). Sections of VYS stained for a4 (green) and F (red) from +/+ embryos (**b**, **c**) and −/− embryos (**d**, **e**). Lysates from VYS of −/− and +/+ embryos (40 μg) and total mouse kidney (MKT) (20 μg) probed with antibodies against various subunits of the proton pump (**f**). β-gal (arrow heads) and H + E counterstained radial section of placenta (**g**) from an e16.5 +/− embryo showing the labyrinthine layer (L), spongiotrophoblast layer (S) or maternal decidual cells (M). High powered image of β-gal/H + E stained maternal tissue (**h**). Western blot of placenta lysates from +/+ embryos, −/− embryos (40 μg) and total mouse kidney (20 μg) blotted with α-a4 and α-C2 (**i**). All scale bars: 20 μm

Lysates from uteri of pregnant and non-pregnant animals subjected to western blot analysis showed the presence of the proton pump in all samples, detected by using the ubiquitous α-D subunit antibody which is a reliable control for western blots. However, differences between non-pregnant and pregnant uteri were observed. In the *Atp6v0a4*^+/−^ (+/−) non pregnant uterus, β-gal staining but not mature a4 protein was detected in the endometrial glands (Additional file 2: Figure S2E, F), which have been implicated in fetal nutrition [29], but neither were present in the uterine luminal lining. In contrast, in pregnant animals the a4 subunit was detected luminally with β-gal staining (Fig. 2 g, h) and also by western blot of lysates (Fig. 2a). These data suggest that changes in the uterine lining lead to changes in the proton pump subunit expression and assembly in this tissue, and support previous reports that expression of *Atp6v0a4* is upregulated along the full length of the uterine luminal epithelium [30]. This underscores the importance of ion transport in implantation and early pregnancy.

The visceral yolk sac (VYS) is a barrier that surrounds the developing conceptus and functions to absorb nutrients from the uterine compartment, process these macromolecules and transport them to the embryo. Nutrients including amino acids are taken up by the trophoectoderm, endocytosed by the VYS [31, 32] and from there transferred directly to the embryonic gut [29]. Immunostaining revealed that both the a4 and F subunits were present in cells of the VYS of +/+ embryos, with a4 being apical but F having a more diffuse staining pattern as expected from its wider expression (Fig. 2b, c respectively). In −/− embryos, a4 was predictably not present but the staining pattern of F remained similar to that seen in +/+ embryos (Fig. 2d, e respectively). Analysis of both +/+ and −/− embryo VYS lysates showed in addition to the a4 subunit, the d2 and C2 subunits were also present, but B1 was absent. The ubiquitous subunit D again provided a positive control (Fig. 2f). RT-PCR from VYS cDNA showed that no G3 or E2 was detected in this tissue type (Additional file 3: Figure S3).

The placental syncytiotrophoblast has been shown to be a location of proton pumps that are thought to facilitate exchange of ions, nutrients and catabolic products between mother and fetus [33]. Placental tissues were harvested between day e15.5 and e17.5 and β-gal activity was seen apically in maternal uterine tissues which surround the placenta, but there was no evidence of staining in any of the distinct layers of the placental disk itself such as the labyrinthine layer, spongiotrophoblast layer or maternal decidual cells (Fig. 2 g, h). a4 protein was also undetectable in placenta total protein; in contrast C2 protein (Fig. 2i), which has previously been shown to be present in the human placenta at RNA level [34], was found.

### Adult male reproductive tract

**Fig. 3:**
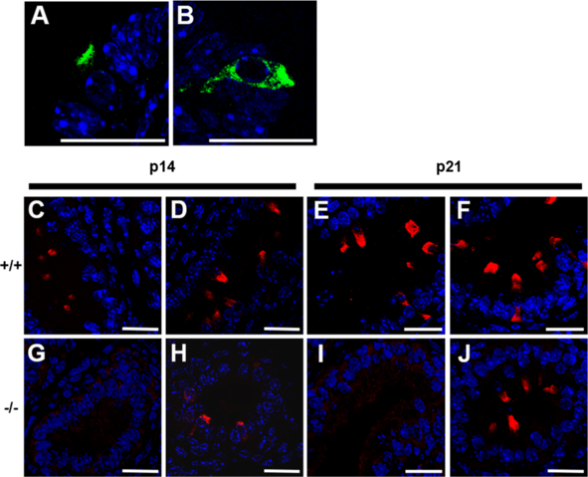
Male reproductive tract. Apical attenuation of the a4 subunit (**a**) and apical attenuation accompanied by intracellular staining for the B1 subunit (**b**) in cells of the ampullary gland. Immunostaining in the epididymis of p14 (**c**, **d**, **g**, **h**) and p21 animals (**e**, **f**, **i**, **j**) showed the a4 subunit was apically expressed in +/+ animals (**c**, **e**) and absent in −/− animals (**g**, **i**). However the B1 subunit’s apical location is similar in both +/+ (**d**, **f**) and −/− animals (**h**, **J**) at both ages. Scale bars: 20 μm

The presence of the proton pump in the male reproductive tract is well established, with non-generic subunits B1, C2, G3, a4, and d2 being reported in the epididymis and vas deferens of rats and mice [2, 17, 35]. In this study, β-gal staining of the whole adult male reproductive tract showed the secretory glands of the preputial glands, which are sebaceous exocrine glands located subcutaneously above the penis, were darkly stained, but the a4 protein itself was not observed (Additional file 4: Figure S4A-C). Seminal vesicles did not show any β-gal staining - similarly the ubiquitous E1 subunit has been reported to be barely detectable by western blot [36]. This is consistent with the absence of large-scale acidification of this segment of the tract, which is relatively alkaline reflecting its role in sperm capacitation. The prostate, although displaying positive β-gal staining, showed no detectable a4 protein when the tissue was subjected to immunostaining (Additional file 4: Figure S4D–F). Transverse sections of genitourinary tract showed rare cells in the paired ampullary glands and prostatic glands having apical a4 and B1 staining (Fig. 3a, b). While these sparse cells are likely to have an acid secreting role, the low number again suggests that there is no large-scale acidification in this segment.

#### The epididymis

The epididymis is the most acidic part of the reproductive tract, with the presence of the proton pump implicated in the maturation of spermatozoa [37]. Immunostaining of epididymides of +/+ mice at p14 and p21 revealed the presence of a4 apically in clear cells, which conforms with previous work showing the appearance of the H^+^ATPase two weeks after birth [38]. To investigate the impact of a4 ablation on pump localisation, epididymides from p14 and p21 +/+ and −/− animals were compared. In +/+ animals a4 and B1 were both seen apically in the narrow/clear cells (Fig. 3c–f) and in −/− animals B1 subunit staining was also observed apically (Fig. 3 h, j), indicating that absence of the a4 subunit does not affect proton pump localisation at either age. Although it has been hypothesised that disruptions in H^+^ATPase activity could affect male fertility due to defective acidification [39, 40], *Atp6v1b1*^*−/−*^ and *Atp6v0a4*^*−/−*^ males are both fertile [21, 41]. Thus, due to the high number of pumps that are observed, and their preserved apical location in the epididymis, it seems likely that a4 activity here is retained by subunit substitution.

## Conclusions

**Fig. 4:**
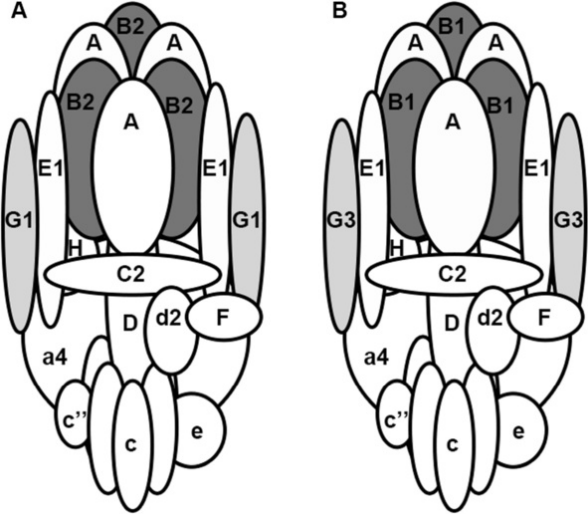
Cartoon of the H^+^ATPase found in visceral yolk sac (**a**) and olfactory epithelium (**b**). The subunits are labelled with capitals for the V_1_ domain and lower case for the V_0_ domain. Differences between the locations are shaded.The schematic is based on the proposed structure of yeast V-ATPases [48] with the third EG dimer not shown

In this study, tissue restricted subunits were identified for the first time in VYS and a model of the pump at this site and in the OE is proposed (Fig. 4a, b) and the expression patterns of these tissue-restricted homologues and others in mice is summarised in Table 1. It has been proposed that the VNO is involved in the detection of non-volatile odorants, which include pheromones [42]. The absence of the B1 and a4 subunits in the VNO of −/− animals would likely lead to the inhibition of odours due to the mucus lining of the duct not having the correct physiological pH, which would result in poor odour absorption and therefore receptor activation. Indeed both *Atp6v1B1*^*−/−*^ and *Atp6v0a4*^*−/−*^ animals have been shown to be hypo-osmic [21, 22], but it was unclear whether this was due to changes in the OE, VNO or both. This study suggests that the murine VNO is the more important segment of the nasal cavity. The reason for the subtle differences in pump subunit combinations is not fully understood, but are thought to account for differences in pump targeting and localisation. The differences in subunit composition could also have implications for regulation of pump assembly and insertion into membranes, and also for the regulatory pathways that control these processes. Dysfunction in some tissue specific pump subunits has been shown to cause a variety of diseases, some of which have been recapitulated in various animal models [14, 21, 41, 43, 44], and a comprehensive knowledge of the subunit combinations may allow for targeted up or down regulation of specific pumps in such disorders.

**Table 1:** Summary of reported H^+^ATPase subunit variants in mouse tissues

Subunit	B	C	E	G	a	d	e
Number of genes	2	2	2	3	4	2	2
Generic Subunit	B2	C1	E1	G1	a1	d1	e1
Kidney	B1	C2		G3	a4	d2	
Epididymis	B1				a4		
Acrosome of sperm			E2		a2		
Ampullary gland and prostatic alveoli	B1*				a4*		
Inner ear	B1				a4		
Nasal Epithelium	B1	C2*		G3*	a4	d2*	
Bone					a3	d2	
Eye					a2, a3, a4		
Lung		C2				*d2*	
Placenta		C2*			a2		
VYS		C2*			a4*	d2*	
Uterus					a4*		

Entries underlined represent protein and RNA evidence, normal entries represent protein evidence and italic entries; RNA evidence. Entries marked with an asterisk are results from this study

## Methods

### Generation and maintenance of Atp6v0a4-deficient mice

Mice generated and genotyped as described previously [21] were housed and reared under standard conditions. All procedures were performed in accordance with the UK Home Office Animals Scientific Procedures Act (1986).

### Preparation of cDNA

Tissues dissected from newly-deceased animals were stored in RNAlater (Qiagen); RNA was then prepared using an RNeasy mini plus kit (Qiagen) and cDNA made using a SuperScript VILO cDNA Synthesis Kit (Invitrogen) according to manufacturer’s instructions. Reverse transcription reactions performed in the absence of SuperScript III Enzyme provided a negative control. cDNA was amplified using primers for a4 [17], G3 [45] and E2 (CCTTCCAATGCTGCTGGAGG and CTCTGCAGTTTAGCCCAGGC, these primers lie in Exon 2 and the 3’-untranslated region respectively, the product does not span any introns) with β-actin as a control to confirm successful reverse transcription.

### Western blot analysis

Mouse kidney total protein lysate were prepared as described previously [3]. 40 μg of each lysate were separated by SDS/PAGE followed by western blot analysis. Primary antibodies used to probe the blots were α-a4 (RA2922, [3]), α-B1 (G-H Sun-Wada [46]), α-D (Santa Cruz sc-21215), α-C2 (raised in chicken against amino acids 9–27 of ATP6V1C2), α-G3 (Abnova H00127124-A01) and/or α-d2 (SK20, [47]) subunits of H^+^ATPase.

### β-galactosidase staining

Fresh mouse postmortem tissues were washed with 0.1 M phosphate buffer (pH 7.3) containing 2 mM MgCl_2_, 0.01 % Nonidet P-40, 0.02 % sodium deoxycholate and stained for β-galalactosidase (β-gal) as described previously [21] then stored in PBS/4 % formaldehyde before being embedded and sectioned.

### Histologic analysis and immunofluorescence

Fresh mouse postmortem tissues fixed in neutral buffer formalin or β-galactosidase stained and then fixed were paraffin-embedded and sectioned to 5 μm. Adult heads were decalcified prior to embedding and sectioning in neutral EDTA [0.34 M EDTA, 0.33 M NaOH, pH 7.06] until soft. After deparaffinization and rehydration through graded ethanol, sections were stained with either H + E, eosin alone or antibody. For immunolocalization, sections were subjected to citrate buffer [10 mM sodium tricitrate, 0.44 mM HCl, 0.05 % Tween-20] antigen retrieval before applying α-a4 (1:1000), α-B1 (1:2000) or α-F (1:500) overnight at 4 °C. Sections were examined using a Zeiss Axioskop2 microscope (for H + E/eosin/β-gal staining) or a Zeiss LMS 510 confocal microscope (for immunostaining).

## Abbreviations

β-gal, β-galactosidase; dRTA, distal renal tubular acidosis; OE, olfactory epithelium; VNO, vomeronasal organ; VYS: visceral yolk sac

## Additional files

Additional file 1: Figure S1. Immunostaining in the vomeronasal organ (VNO). High powered images of a4, B1 and F staining in the VNO of p5 +/+ animals (A, B, D respectively) compared to B1 and F staining in −/− animals (C and E respectively). Scale bars: 20 μm. (DOCX 183 kb) Additional file 2: Figure S2. a4 expression in the female reproductive tract. Schematic of the female murine reproductive tract; each ovary (1) is attached to a uterine horn (2) which are joined at the fundus to form a Y shape. From the fundus the cervix (3) leads down to the vagina (4). (A). β-gal activity was present in the stratified squamous epithelium of the cervical canal (B, black arrowheads) but immunostaining showed a4 was not detected (C) and F had no apical attenuation (D). β-gal activity was detected in the endometrial glands of the non-pregnant uterus (E, white arrowheads) but not observed in the uterine lumen (E, black arrow head); when the uterine glands were immunostained a4 protein was not detectable (F, white arrow heads). Scale bars: 20 μm. (DOCX 1596 kb) Additional file 3: Figure S3. RT-PCR amplification of the G3 and E2 subunit isoforms from mouse visceral yolk sac. PCR amplification of cDNA from 3 visceral yolk sac samples (+) with respective RT- controls (−), negative PCR control (C-) and positive PCR control of genomic DNA (for actin and E2) or kidney cDNA (G3) (C+). (DOCX 40 kb) Additional file 4: Figure S4. H+ATPase expression in the male reproductive tract. (A) Coronal section of the preputial gland showing β-gal activity in the secretory glands, immunostaining do not detect any a4 protein (B) and the F subunit (C) had no apical attenuation. The lateral prostate displayed β-gal activity but the a4 protein was not detected by immunostaining (E) and the F subunit was intracellular. Scale bars: 20 μm. (DOCX 1264 kb)
