# Supplementary material for: Extra-renal locations of the a4 subunit of H+ATPase
Source: BMC Cell Biol. 2016 Jul 2;17:27. doi: 10.1186/s12860-016-0106-8 (PMC4930620; doi:10.1186/s12860-016-0106-8)
Supplement: Additional file 1: Figure S1. — Immunostaining in the vomeronasal organ (VNO). High powered images of a4, B1 and F staining in the VNO of p5 +/+ animals (A, B, D respectively) compared to B1 and F staining in −/− animals (C and E respectively). Scale bars: 20 μm. (DOCX 183 kb) [file 12860_2016_106_MOESM1_ESM.docx]

Supplementary Figure 1


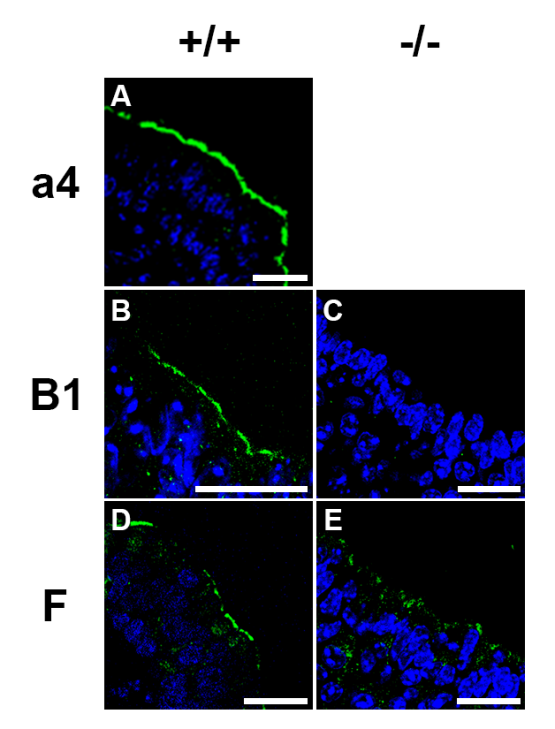


Immunostaining in the vomeronasal organ (VNO). High powered images of a4, B1 and F staining in the VNO of p5 +/+ animals (A, B, D respectively) compared to B1 and F staining in -/- animals (C and E respectively). Scale bars: 20µm
