# Supplementary material for: Extra-renal locations of the a4 subunit of H+ATPase
Source: BMC Cell Biol. 2016 Jul 2;17:27. doi: 10.1186/s12860-016-0106-8 (PMC4930620; doi:10.1186/s12860-016-0106-8)
Supplement: Additional file 2: Figure S2. — a4 expression in the female reproductive tract. Schematic of the female murine reproductive tract; each ovary (1) is attached to a uterine horn (2) which are joined at the fundus to form a Y shape. From the fundus the cervix (3) leads down to the vagina (4). (A). β-gal activity was present in the stratified squamous epithelium of the cervical canal (B, black arrowheads) but immunostaining showed a4 was not detected (C) and F had no apical attenuation (D). β-gal activity was detected in the endometrial glands of the non-pregnant uterus (E, white arrowheads) but not observed in the uterine lumen (E, black arrow head); when the uterine glands were immunostained a4 protein was not detectable (F, white arrow heads). Scale bars: 20 μm. (DOCX 1596 kb) [file 12860_2016_106_MOESM2_ESM.docx]

Supplementary Figure 2


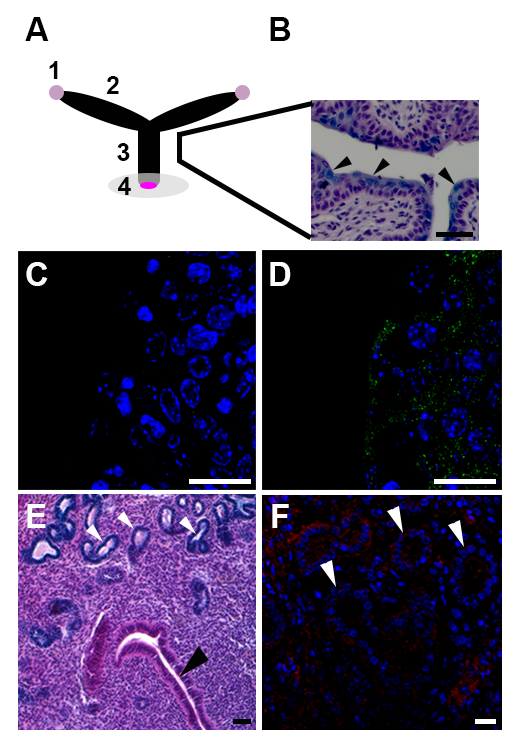


Schematic of the female murine reproductive tract; each ovary (1) is attached to a uterine horn (2)

which are joined at the fundus to form a Y shape. From the fundus the cervix (3) leads down to the

vagina (4) (A). β-gal activity was present in the stratified squamous epithelium of the cervical canal (B, black arrowheads) but immunostaining showed a4 was not detected (C) and F had no apical attenuation (D). β-gal activity was detected in the endometrial glands of the non-pregnant uterus (E, white arrowheads) but not observed in the uterine lumen (E, black arrow head); when the uterine glands were immunostained a4 protein was not detectable (F, white arrow heads). Scale bars: 20 μM
