# Supplementary material for: Extra-renal locations of the a4 subunit of H+ATPase
Source: BMC Cell Biol. 2016 Jul 2;17:27. doi: 10.1186/s12860-016-0106-8 (PMC4930620; doi:10.1186/s12860-016-0106-8)
Supplement: Additional file 3: Figure S3. — RT-PCR amplification of the G3 and E2 subunit isoforms from mouse visceral yolk sac. PCR amplification of cDNA from 3 visceral yolk sac samples (+) with respective RT- controls (−), negative PCR control (C-) and positive PCR control of genomic DNA (for actin and E2) or kidney cDNA (G3) (C+). (DOCX 40 kb) [file 12860_2016_106_MOESM3_ESM.docx]

Supplementary Figure 3


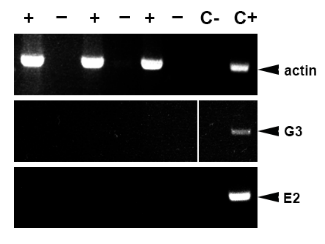


RT-PCR amplification of the G3 and E2 subunit isoforms from mouse visceral yolk sac. PCR amplification of cDNA from 3 visceral yolk sac samples (+) with respective RT- controls (-), negative PCR control (C-) and positive PCR control of genomic DNA (for actin and E2) or kidney cDNA (G3) (C+).
