# Supplementary material for: Extra-renal locations of the a4 subunit of H+ATPase
Source: BMC Cell Biol. 2016 Jul 2;17:27. doi: 10.1186/s12860-016-0106-8 (PMC4930620; doi:10.1186/s12860-016-0106-8)
Supplement: Additional file 4: Figure S4. — H+ATPase expression in the male reproductive tract. (A) Coronal section of the preputial gland showing β-gal activity in the secretory glands, immunostaining do not detect any a4 protein (B) and the F subunit (C) had no apical attenuation. The lateral prostate displayed β-gal activity but the a4 protein was not detected by immunostaining (E) and the F subunit was intracellular. Scale bars: 20 μm. (DOCX 1264 kb) [file 12860_2016_106_MOESM4_ESM.docx]

Supplementary Figure 4


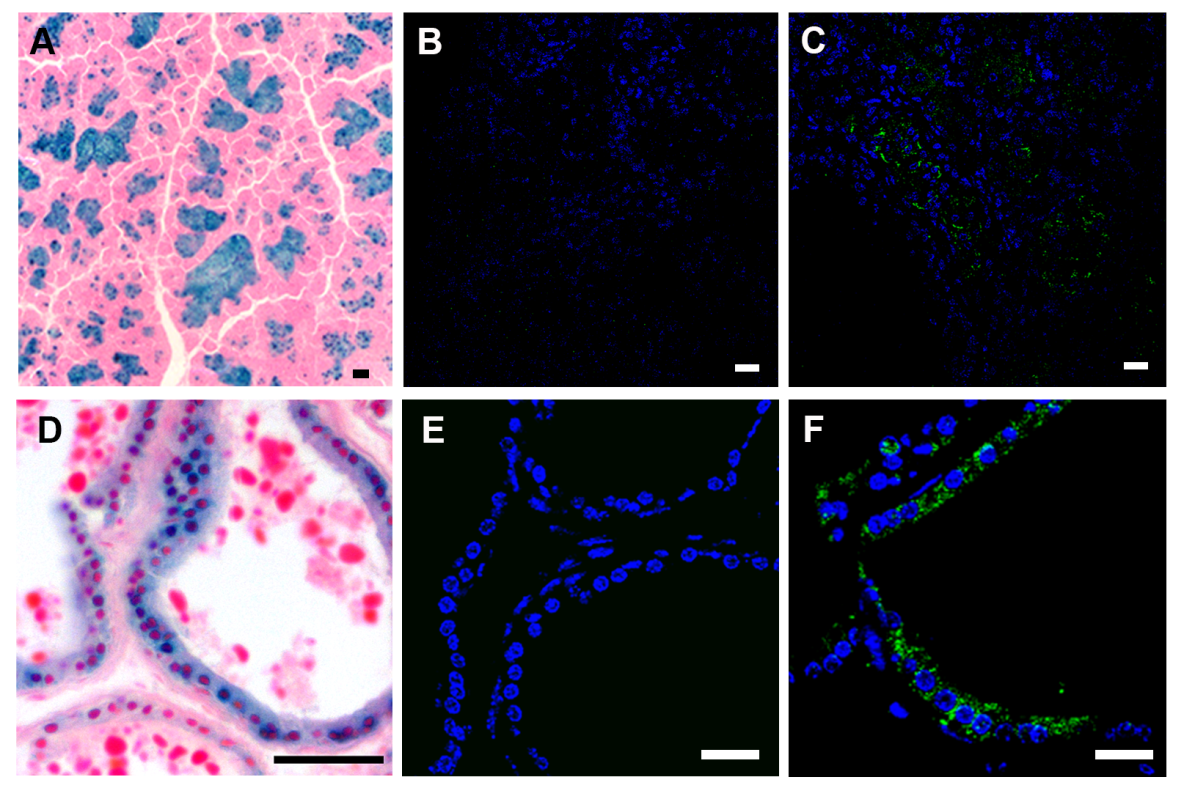


Coronal section of the preputial gland showing β-gal activity in the secretory glands, immunostaining do not detect any a4 protein (B) and the F subunit (C) had no apical attenuation. The lateral prostate displayed β-gal activity but the a4 protein was not detected by immunostaining (E) and the F subunit was intracellular. Scale bars: 20 μM
